# Artificial intelligence-based machine learning models for preoperative diagnosis and staging of ovarian tumors

**DOI:** 10.1186/s43046-025-00337-4

**Published:** 2026-01-05

**Authors:** Soheila Aminimoghaddam, Hamid Mokhtari Torshizi, Roghayeh Pourali, Arash Mohazzab

**Affiliations:** 1 Department of Obstetrics and Gynecology, School of Medicine, Firoozgar Hospital, Iran University of Medical Sciences, Karimkhan Zand Boulevard, Behafarin St, Tehran, Iran; 2 Biomedical Engineering and Physics Department, School of Medicine, Shahid Beheshti University of Medical Sciences, Tehran, Iran; 3 Department of Epidemiology, School of Public Health, Iran University of Medical Sciences, Tehran, Iran

**Keywords:** Ovarian cancer, Ovarian mass, Machine learning, Artificial intelligence

## Abstract

**Background:**

Ovarian cancer remains the most lethal gynecological malignancy, necessitating precise diagnostic strategies to improve patient outcomes. This study aims to develop and evaluate machine learning models that utilize patient history, imaging, and blood test data to differentiate between benign and malignant ovarian tumors and predict the stage of malignant cases.

**Methods:**

A total of 357 individuals diagnosed with ovarian tumors participated in the study. Among these, 139 tumors were identified as benign, 40 as borderline, and 178 as malignant. The analysis employed four machine learning classifiers support vector machine (SVM), random forest (RF), logistic regression (LR), and decision tree utilizing 39 features derived from blood tests, imaging, and the patient’s background to generate diagnostic outcomes. The study focused on assessing the significance of these features in predicting malignancy and determining the stage of the disease.

**Results:**

The RF algorithm demonstrated the highest accuracy, reaching 94% based on imaging and tumor markers, with an AUC of 0.9. Key features contributing to this success include Human Epididymis Protein 4 (HE4) and Cancer Antigen 125 (CA125). In terms of staging malignant tumors, the SVM exhibited lower error rates, particularly in predicting advanced-stage disease (AUC: 0.77). Notably, CA125 and the presence of ascites emerged as the most influential factors for accurately staging the disease.

**Conclusion:**

The utilization of AI models proves effective in accurately classifying both malignant and benign ovarian tumors, showcasing promising advancements in diagnostic capabilities.

## Introduction

Ovarian tumors are frequently observed in gynecology, presenting as either benign, malignant, or borderline masses. The lifetime risk of ovarian cancer for women is 1.3%, with an annual incidence of 6.6 cases per 100,000 women. Notably, the likelihood of malignant ovarian tumors escalates from 13% in pre-menopausal age to 45% in post-menopausal age [1]. The approach to managing these masses across various age groups is contingent on factors such as mass size, patient age, fertility preservation goals, potential malignancy, and the manifestation of symptoms [2]. To classify ovarian tumors, imaging techniques like Ultrasound, Computed Tomography (CT) scans, Magnetic Resonance Imaging (MRI), and serum biomarkers are employed. The ORADS system (Ovarian-Adnexal Reporting and Data System) has been established to minimize heterogeneity and ambiguity in different imaging reports [3]. Additionally, certain biomarkers prove useful. The Risk of Malignancy Algorithm (ROMA) is utilized to assess the likelihood of malignancy in ovarian tumors based on serum levels of CA125 (Cancer Antigen 125), HE4 (Human Epididymis Protein 4), and menopausal status [4].

Contemporary medical science has witnessed significant progress due to the adoption of new technologies, with the Medical Decision Support System (DSS) standing out as a notable example [5]. In recent years, artificial intelligence (AI) networks have emerged as innovative tools alleviating diverse medical challenges. These AI networks, alongside machine learning algorithms, play a crucial role in diagnosing, researching, and treating cancers, including breast cancer, gastrointestinal cancer, and lung cancer [6, 7]. Gynecological cancers, particularly ovarian carcinoma, have been subjects of various studies exploring computer-aided methods [8, 9]. Notably, automated analysis of digital colposcopic images has been extensively studied for diagnosing cervical cancer [10]. Given the lethality of ovarian carcinoma as the most lethal gynecological cancer [11], ensuring accurate diagnosis of ovarian tumors becomes imperative. However, the risk of over-diagnosis leading to unnecessary and burdensome surgeries on patients cannot be ignored. Several studies on the sonographic evaluation of ovarian tumors indicate that AI and regression models can effectively differentiate between benign and malignant tumors. The implementation of these systems holds the potential to avert unnecessary interventions, thereby mitigating patient harm and reducing costs.

The objective of this study is to employ AI models utilizing patient history, imaging, and blood tests for the purpose of distinguishing between benign and malignant ovarian tumors. Furthermore, the study aims to predict the stage of the disease specifically in malignant tumors.

## Patients and methods

### Patients and features

This cross-sectional study was conducted at Tehran’s Firozgar Hospital, affiliated with the Iran University of Medical Sciences. Following approval from the ethics committee (code: IR.IUMS.FMD.REC.1401.638), data were collected using the hospital’s information registration system. In cases of digital data gaps, paper files were referenced. The study focused on patients who underwent surgery for ovarian tumors between 2012 and 2022, with 357 out of 468 eligible patients enrolled. Pathological analysis revealed 139 benign tumors, 40 borderline cases, and 178 malignant tumors.

Inclusion criteria encompassed all patients who underwent surgery for ovarian tumors, with the final diagnosis based on pathology, while exclusion criteria involved the absence of clinical or paraclinical data. Each patient contributed 37 features, which were categorized into four groups based on the data source to delineate factors influencing different learning algorithms:

1. Ultrasonographic characteristics of the mass (size, unilateral or bilateral, presence of thick septa, increased vascularity in color Doppler, decreased vascular resistance, presence of papillary projection, irregular border, solid component, ascites, involvement of omentum). The ultrasound examinations were carried out using a Philips Affiniti 50 system (Philips Healthcare, Eindhoven, The Netherlands) equipped with a convex/curvilinear transducer typically operating at frequency range 2–9 MHz for pelvic imaging.
2. Tumor markers (CA125, Carcino Embryonic Antigen (CEA), Alfa Feto Protein (AFP), Risk of Malignancy Algorithm (ROMA), Cancer Antigen 19 − 9 (CA19-9), HE4, beta Human Chorionic Gonadotropin (BHCG), Lactate Dehydrogenase (LDH), CA125/CEA).
3. Patient’s background (age, history of gastrointestinal or gynecological cancer in the patient’s first-degree family, personal history of cancer, history of infertility, history of cesarean section, history of endometriosis, gastrointestinal symptoms, urinary symptoms, menopause status, gravidity, parity, history of oral contraceptive (OC) use, diagnosis of mass during pregnancy).
4. Complete blood count (CBC) data (hemoglobin (Hb) level, white blood cell count (WBC), lymphocyte percentage, neutrophils percentage, and platelets).

The ultrasound-measured maximum diameter determined the size of the mass. Endometriosis history was documented based on patient reports. Thick septa were identified when septa measured ≥ 3 millimeters in ultrasound. In malignant tumors, stage I denoted the early stage, while stages II-IV were categorized as advanced stages. The histological classification of tumors followed the 2020 WHO classification for female genital tract tumors [12].

### Statistical analysis

The data are presented as mean (SD). Parametric variables were compared among groups using a one-way ANOVA (Analysis of Variances) test, followed by pairwise comparisons through the Bonferroni post-hoc test. For non-parametric quantitative variables, the Kruskal-Wallis one-way analysis of variance test was employed. Categorical variables were compared using the chi-square test. Statistical analyses were conducted using SPSS Software version 23 (IBM Company, NY), with a significance level set at 0.05.

### AI models and evaluation techniques

Four machine learning classifiers, namely support vector machine (SVM), random forest (RF), logistic regression (LR), and decision tree, were employed to extract diagnostic outcomes from the previously mentioned 37 features.

In supervised learning, categorization is a fundamental component. With a training dataset comprising observations and corresponding class outputs, the objective of classification is to acquire a general rule mapping features to their correct categories. Given our binary classification system in the statistical population, divided into two categories, we focus on binary classification. Considering the limited sample size of the borderline tumor group, a comparison was conducted between malignant and benign masses. A total of 317 patients were randomly assigned to either “training” (80%) or “testing” (20%) data sets.

To enhance robustness, we implemented the cross-validation algorithm. In each iteration, 20% of the population was randomly selected for testing, and 80% for training. This process was repeated 10 times. We evaluated the performance using the confusion matrix, accuracy, f1 score, and area under the curve as numerical metrics, alongside the ROC curve as an intuitive scale.

$$\:\mathrm{precision=\:}\frac{\mathrm{TP}}{\mathrm{TP+FP}}$$

$$\:\mathrm{Recall=\:}\frac{\mathrm{TP}}{\mathrm{TP+FN}}$$

$$\:\mathrm{f1=\:}\frac{\mathrm{2*(precision*Recall)}}{\mathrm{precision+Recall}}$$

## Results

This study comprised 357 participants, with pathology results indicating 139 benign tumors, 40 borderline cases, and 178 malignant tumors. Table 1 presents the clinical and paraclinical characteristics of individuals across the three groups. The average age of the patients was 45.97 years. Eight pregnant participants were excluded from the BHCG analysis. All participants reported experiencing gastrointestinal symptoms, such as varying degrees of abdominal pain, abdominal enlargement, nausea, vomiting, or changes in bowel habits.

**Table 1:** Data set of 357 patients

	Benign		Borderline		Malignant		
	*N* = 139		*N* = 40		*N* = 178		
Feature	Frequency	Percent	Frequency	Percent	Frequency	Percent	*P*-value
Bilateralal mass	17	9.6	3	6.7	32	16.7	0.017
Thick Septa	57	32.2	25	55.6	92	47.9	0.001
Hypervascularity	5	2.6	3	6.7	38	19.8	< 0.001
Hyporesistance	0	0	0	0	9	4.7	0.005
Papillary Projection	18	10.2	13	28.9	10	5.2	< 0.001
Solid component	57	32.2	18	40	129	67.2	< 0.001
Irregular border	13	7.3	8	17.8	14	7.3	0.059
Cancer personal Hx	1	1.4	1	2.5	11	6.2	0.086
GYN cancer FH	1	0.6	1	2.2	7	3.6	0.008
Omental involvementent	0	0	2	4.4	36	18.8	< 0.001
Infertility	7	4	1	2.2	10	5.2	0.67
Cesarean section	60	33.9	15	33.3	57	29.7	0.428
Urinary symptoms	9	5.1	3	6.7	21	10.9	0.11
Menopause	50	28.2	12	26.7	88	45.8	0.001
OC	9	5.1	1	2.2	4	2.1	0.253
Endometriosis	4	2.87	0		2	1.12	0.329
Acites	4	2.87	9	22.5	99	55.61	< 0.001

	Mean	SD	Mean	SD	Mean	SD	
Age(years)	43.890	15.398	41.800	14.934	48.530*	13.135*	0.003
Gravidity	2.740	2.510	2.500	2.900	3.066	2.660	0.366
Parity	2.400	2.280	2.150	2.720	2.700	2.410	0.328
Size (mm)	77.310*⁑	42.450	97.810*	47.250	98.720⁑	51.340	< 0.001
Hb(g/dl)	12.699*	1.519	12.838⁑	1.574	11.939*⁑	1.677	< 0.001
WBC(wbc/mcl)	7641.010*	2104.819	8190.000	2114.638	8444.380*	3340.438	0.04
Platelet(platelate/mcl)	292381.290	290559.216	264025.000	79961.686	321786.520	119471.148	0.183
CA125(unit/ml)	41.472*⁑	108.944	704.717*	3484.467	618.751⁑	1289.656	0.001
CA19-9(unit/ml)	20.423*	20.453	572.193*⁑	3221.057	108.539⁑	711.600	0.034
CEA(ng/ml)	2.791*	6.091	6.154	15.049	8.573*	26.469	0.019
AFP(ng/ml)	2.507	3.003	4.103	7.816	447.577	2882.146	0.1
ROMA	7.428*⁑	10.630	24.548*‡	23.117	46.347⁑‡	35.430	0.009
HE4(pmol/l)	54.258*	22.320	84.530⁑	62.625	203.412*⁑	243.347	< 0.001
Lymphocyte(percent)	0.300	0.098	0.263	0.089	0.249	0.089	< 0.001
Neutrophil(percent)	0.626*	0.104	0.669	0.102	0.674*	0.100	< 0.001
BHCG ^3^ (miu/ml)	1.697	1.238	1.149	1.379	9.708	76.618	0.382
CA125/CEA	30.287*⁑	54.571	799.072*	3894.595	589.186⁑	1361.009	0.002
LDH(u/l)	395.885*	153.689	411.125⁑	165.740	655.443*⁑	439.426	< 0.001

1. Categorical variables were compared using chi squared test, Quantitative variables were compared by ANOVA and Kruskal Wallis test

2. Pairwise comparison was done by Bonferroni post-hoc test and significant pair wise differences was marked by same symbol

3. We excluded the 8 patients who were pregnant from the analysis of BHCG

4. *AFP* Alpha Feto Protein, *BHCG* Beta Human Chorionoc Gonadotropin, *CA125 *Cancer Antigen125,CA19-9 Cancer Antigen19-9, *CEA* CarcinoEmbryonic Antigen, *FH* Family History, *Hb* Hemoglobin, *HE4* Human Epididymis Protein4, *HX* History, *LDH* Lactate DeHydrogenase, *mm* Millimeter, *OC* Oral Contraceptive, *ROMA* Risk of Malignancy Algorithm, *WBC* White Blood Cell

Gravidity and parity do not differ among the three groups, with an average parity of 2.52 for all patients. However, significant differences exist in various variables among the three groups, including size, bilaterality, presence of thick septa, increased vascularity in color Doppler, decreased vascular resistance, presence of papillary projection, irregular border, solid component, ascites, omentum involvement, CA125, CEA, ROMA index, CA19-9, HE4, LDH, CA125/CEA, age, history of digestive disorders in the patient’s first-degree family, menopause status, hemoglobin level, white blood cell count, lymphocyte percentage, and neutrophil percentage.

In the malignant group, there is a higher proportion of menopausal patients (45.8%). Surprisingly, borderline tumors exhibit higher average levels of CA125 and CA19-9 than other tumors, while average HE4 and LDH are significantly higher in malignant tumors. Additionally, the malignant group shows a lower average hemoglobin level. This study employed four machine learning algorithms—SVM, RF, LR, and decision tree. Due to the small size of the borderline tumors group, we compared 317 patients with malignant and benign tumors. Each category of features was separately used to train the four different algorithms.

Table 2 presents the outcomes of all models for malignancy prediction in ovarian tumors. When trained on imaging data, the LR algorithm demonstrated the highest accuracy (0.86 AUC: 0.86), while the accuracy of the three RF algorithms, SVM, and LR were nearly equal.

**Table 2:** Results of all models for predicting malignancy in ovarian tumors

Data	Algorithm	Accuracy	Precision	Recall	F1	AUC
	Decision Tree	0.70	0.69	0.72	0.70	0.71
Image	SVM	0.86	0.87	0.83	0.85	0.85
	Random Forest	0.85	0.88	0.76	0.82	0.80
	LR	0.86	0.82	0.86	0.84	0.86
	Decision Tree	0.60	0.60	0.50	0.54	0.59
CBC	SVM	0.64	0.65	0.47	0.55	0.62
	Random Forest	0.66	0.68	0.52	0.59	0.64
	LR	0.72	0.70	0.57	0.63	0.69
	Decision Tree	0.50	0.47	0.29	0.35	0.49
Patient’s background	SVM	0.52	0.56	0.34	0.42	0.52
	Random Forest	0.49	0.52	0.52	0.52	0.52
	LR	0.56	0.50	0.59	0.54	0.56
	Decision Tree	0.82	0.83	0.79	0.80	0.81
Tumor markers	SVM	0.80	0.83	0.68	0.74	0.78
	Random Forest	0.90	0.81	0.84	0.83	0.81
	LR	0.82	0.82	0.70	0.75	0.80
	Decision Tree	0.84	0.81	0.81	0.81	0.83
Tumor markers +	SVM	0.82	0.86	0.83	0.84	0.81
Image	Random Forest	0.94	0.95	0.84	0.90	0.90
	LR	0.84	0.86	0.80	0.83	0.84

**Fig. 1:**
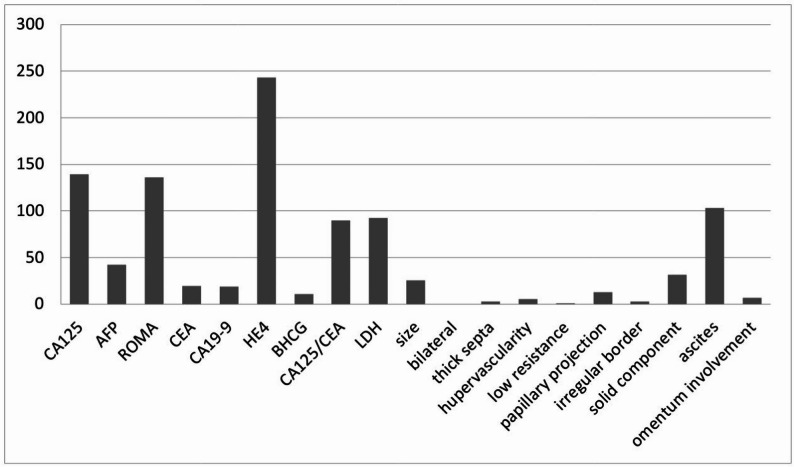
Comparison of feature importance in predicting malignancy : HE4, CA125,ROMA index and Ascites are the best predicting factors

Upon utilizing a combination of imaging and tumor marker data, the RF algorithm achieved the highest accuracy at 0.94 (AUC: 0.9), followed by 0.84 for the decision tree and LR, and 0.83 for SVM. The LR algorithm achieved the highest accuracy based solely on image data at 0.86 (AUC: 0.86). Feature importance in predicting malignancy is illustrated in Fig. 1, with HE4, CA125, ROMA index, and ascites identified as the most significant predictors.

**Fig. 2:**
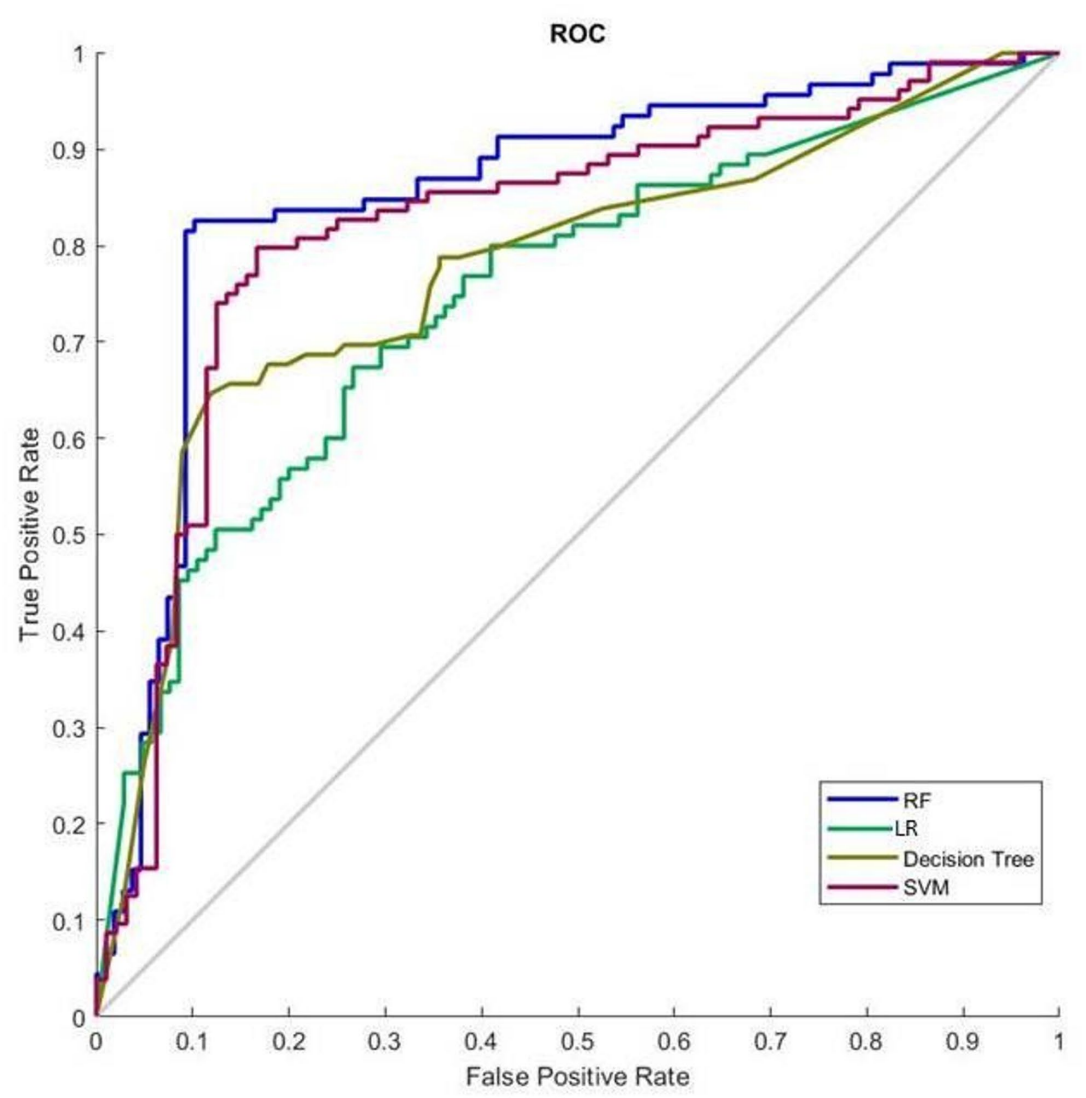
Roc curves derived from RF, LR, Decision Tree and SVM. SVM: support vector machine, RF: random forest, LR: logistic regression

Figure 2 displays the ROC results for each algorithm, indicating that the RF model exhibited the best accuracy (AUC: 0.9) in predicting malignancy in ovarian tumors.

Another objective of this study was to predict the disease stage of malignant tumors, categorizing them into advanced and early stages. At the time of diagnosis, 99 patients (55.6%) were in the advanced stage, and 68 patients (38.2%) were in the early stage. In staging malignant tumors, the SVM algorithm demonstrated the highest precision in all categories (Table 3).

**Table 3:** Results of all models for predicting stage of disease in ovarian cancer

Data	Algorithm	Accuracy	Precision	Recall	F1	AUC
	Decision Tree	0.75	0.71	0.90	0.80	0.73
	SVM	0.65	0.62	0.55	0.58	0.64
Image	Random Forest	0.83	0.88	0.72	0.79	0.80
	LR	0.80	0.81	0.81	0.81	0.79
	Decision Tree	0.75	0.85	0.80	0.82	0.70
Tumor markers	SVM	0.75	0.80	0.72	0.76	0.75
	Random Forest	0.89	0.76	0.83	0.80	0.72
	LR	0.70	0.72	0.72	0.72	0.69
	Decision Tree	0.75	0.70	0.77	0.73	0.75
Tumor markers+	SVM	0.85	0.90	0.83	0.86	0.85
Image	Random Forest	0.91	0.86	0.92	0.89	0.80
	LR	0.75	0.72	0.80	0.76	0.75

**Fig. 3:**
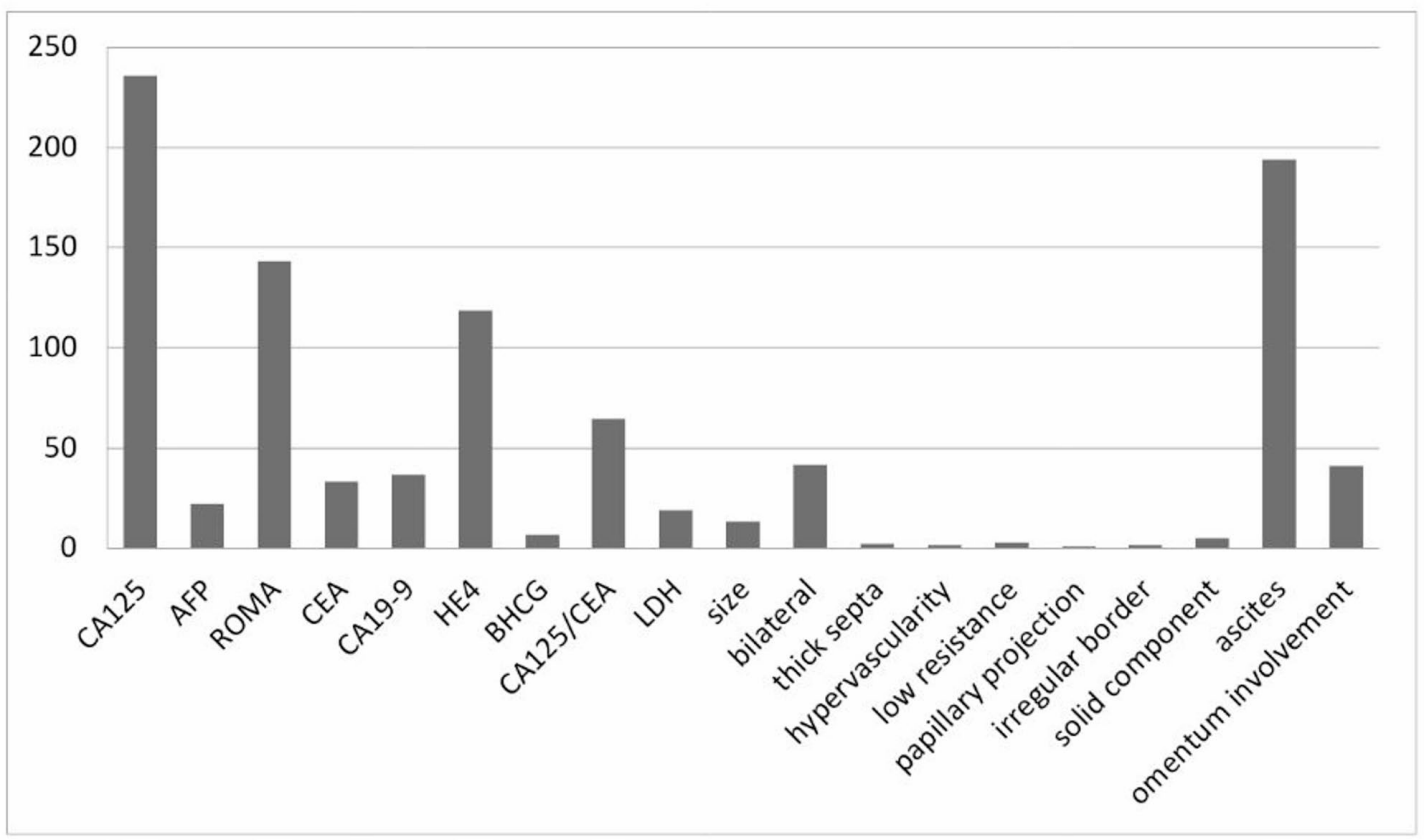
Comparison of feature importance in predicting stage of disease : CA125, Ascites and ROMA index are the best predicting factors

Conversely, the RF algorithm exhibited higher recall than SVM. Consequently, SVM showed less error in differentiating the advanced stage, while the RF algorithm had less error in differentiating the early stage. The highest accuracy in this context was 0.91 in the RF algorithm (AUC: 0.8) with combined imaging and tumor markers data. CA125, ascites, and ROMA index emerged as the most crucial features in predicting the stage of the disease (Fig. 2). Fig. 3 illustrates the relative importance of features in predicting the stage of malignant ovarian tumors. The most significant predictive factors were CA125, ascites, and the ROMA index. The presence of ascites played a crucial role in differentiating early from advanced stages, indicating its strong association with peritoneal spread. The CA125 marker exhibited a robust correlation with higher disease burden, reinforcing its utility in staging. The ROMA index, integrating menopausal status with CA125 and HE4 levels, emerged as another key determinant in classification. These findings underscore the potential of AI models to aid clinicians in preoperative staging, reducing reliance on invasive procedures.

## Discussion

Ovarian tumors present a diagnostic challenge. While preoperative imaging and blood tests can suggest suspicion of malignancy, accurate diagnosis and determination of the disease stage still require surgical resection and histopathological examination. It is imperative to achieve a precise diagnosis to avoid unnecessary surgical interventions in benign masses and accelerate the diagnosis and treatment in cases of malignancy.

The utilization of AI technology and machine learning systems in the medical field has witnessed a notable rise in recent years. Numerous studies have explored the application of these systems in the diagnosis, prediction, and treatment of various diseases. In gynecological oncology, these technologies find prominent use, particularly in cervical and ovarian cancer, with the aim of enhancing screening procedures and tailoring personalized therapies [8]. AI, serving as an alternative to traditional statistical models, proves valuable in identifying non-linear relationships between diverse variables and predicting patterns [13]. Advancements in AI and machine learning have improved cancer diagnostics by integrating genomics, pathology, and radiology data. These models enhance tumor classification, predict behavior, and aid clinical trial design through synthetic data like digital twins [14].

This study involved the training of four distinct AI algorithms. In predicting malignancy, the RF algorithm demonstrated the highest accuracy at 0.94 (AUC: 0.9) when utilizing combined imaging and tumor markers categories. For predicting the disease stage in malignant tumors, the SVM algorithm exhibited the highest precision. In this setting, the RF algorithm achieved the highest accuracy of 0.91 (AUC: 0.8) when employing combined imaging and tumor markers data.

Mihaela Grigere’s review study on the sonographic assessment of ovarian tumors has indicated that both AI and regression models can proficiently distinguish between benign and malignant tumors [15]. Recently, using a dataset of over 17,000 ultrasound images from multiple centers across eight countries, a study validated transformer-based neural networks that significantly outperformed both expert and non-expert sonographers in distinguishing benign from malignant ovarian tumors. Notably, AI-assisted triage reduced unnecessary expert referrals by 63%, addressing the global shortage of experienced ultrasound examiners [16].

D. Timmerman and team conducted research on distinguishing between benign and malignant ovarian tumors before surgery using an artificial intelligence network (ANN). The first network, with a probability of malignancy exceeding 45%, exhibited a sensitivity of 97% and specificity of 89.2%. In cases with a likelihood of malignancy exceeding 60%, the second network demonstrated 97% sensitivity and 92.8% specificity. These results were derived from considerations of age, reproductive status, ultrasound findings, and CA125 levels [17].

Munetoshi Akazawa and Kazunori Hashimoto explored the effectiveness of AI models in distinguishing various types of ovarian tumors. The XG Boost algorithm exhibited the highest diagnostic accuracy at 0.80, followed by 0.78 for RF, 0.67 for LR, and 0.62 for the SVM algorithm. Regarding tumor markers, CA125 levels were found to be highest in malignant masses. When comparing the correlation coefficients of each characteristic, albumin, ascites, and CRP emerged as the most reliable predictive factors [18].

Eiryo Kawakami and colleagues investigated the application of AI in the preoperative diagnosis and prognostic prediction of ovarian epithelial cancers using 32 blood biomarkers. The GBM model achieved the highest diagnostic accuracy at 93.7% (AUC 0.976), followed by SVM with an accuracy of 90.5% (AUC 0.939). They employed Ordinal classification to predict the cancer stage based on preoperative findings, obtaining an accuracy of 69% with an AUC of 0.76 [19].

In their study, Lu et al. assessed 49 demographic and laboratory variables and determined that constructing a decision tree model with the two biomarkers HE4 and CEA was both simple and practical. Their model exhibited an accuracy of 0.95 in predicting ovarian cancer, and its performance was comparable to that of ROMA [20].

According to our findings, when we trained the algorithms using imaging data, the LR algorithm demonstrated the highest accuracy (0.86 AUC: 0.86), and the accuracy of the three RF, SVM, and LR algorithms was nearly identical. In the imaging plus tumor markers category, the RF algorithm achieved the highest accuracy (0.94). Given that precision was found to have a lower value than recall in this context, false positives (FP) held more significance than false negatives (FN).

The efficacy of the RF algorithm arises from combining diverse trees. In this methodology, the ultimate decision, determined through majority voting, diminishes high variance. Employing 100 decision tree designs, the gini optimization algorithm, and min_samples_split = 30, the RF algorithm produced optimal results. Remarkably, HE4, CA125, ROMA index, and ascites stood out as the most influential predictive features. Conversely, CBC data and patient characteristics exhibited limited value in predicting malignancy in this study.

Concerning the staging of malignant tumors, the SVM algorithm exhibits higher precision compared to the RF algorithm. Conversely, the RF algorithm demonstrates higher recall than SVM. Consequently, SVM shows less error in distinguishing the advanced stage, while the RF algorithm has less error in distinguishing the early stage. In predicting the disease stage, the best predictive features are CA125, ascites, and the ROMA index.

In Enshaei et al.‘s study, the ANN demonstrated an impressive 93% accuracy in predicting patients’ survival (AUC = 0.74). Additionally, this model achieved a 77% accuracy in predicting the outcome of surgery, specifically distinguishing between complete debulking and suboptimal cytoreduction [21]. We did not evaluate these results due to a lack of surgical and survival data in most patients.

In summary, the objective of this investigation was to assess the capability of AI models in predicting malignancy in ovarian tumors preoperatively. The findings indicate that AI algorithms can classify ovarian tumors with satisfactory accuracy, and our results align closely with those reported in other studies. However, certain limitations need acknowledgment. This study was carried out in a single oncology center, and the sample size, particularly in the borderline tumors group, was relatively small. Additionally, due to a lack of data, we were unable to explore whether these factors could contribute to predicting disease recurrence. Further research with an expanded sample size and comprehensive patient follow-up could provide more conclusive insights. This study was conducted in a referral educational hospital where both attending gynecologic sonographers and senior residents routinely performed pelvic ultrasound examinations. Because the operator of each examination was not consistently documented in the patient records, we could not analyse our data according to examiner. Consequently, inter-observer variability may have introduced measurement bias and should be considered a limitation of this study.

## Conclusions

This study demonstrates that artificial intelligence–driven machine learning models can meaningfully improve the preoperative assessment of ovarian tumors. In particular, the Random Forest algorithm showed the highest accuracy for distinguishing malignant from benign tumors, while the Support Vector Machine provided the best precision for staging malignant disease. These results suggest that AI-based diagnostic tools could complement conventional imaging and biomarker evaluation to support earlier and more accurate surgical planning and individualized treatment strategies.

The findings also highlight important opportunities for future work. Prospective, multi-center studies with larger and more diverse patient populations are needed to validate these models and to ensure their generalizability across different clinical settings. Further research should also focus on integrating longitudinal data—such as follow-up imaging, treatment response, and recurrence outcomes—to develop models that not only classify and stage disease but also predict recurrence risk and guide therapy selection. Ultimately, incorporating robust, externally validated AI models into routine gynecologic oncology practice could reduce unnecessary surgery, improve risk stratification, and optimize patient management.

## Data Availability

The datasets analyzed during the current study are not publicly available but are available from the corresponding author on reasonable request.

## Abbreviations

AUC: Area Under the Curve
BHCG: Beta Human Chorionic Gonadotropin
CA: Cancer Antigen
CBC: Complete Blood Count
CEA: Carcino-Embryonic Antigen
CRP: C-Reactive Protein
CT: Computed Tomography
DSS: Decision Support System
GBM: Gradient Boosting Machine
Hb: Hemoglobin
HE4: Human Epididymis Protein 4
LDH: Lactate Dehydrogenase
LR: Logistic Regression
MRI: Magnetic Resonance Imaging
OC: Oral Contraceptive
ORADS: Ovarian-Adnexal Reporting and Data System
RF: Random Forest
ROMA: Risk of Malignancy Algorithm
SVM: Support Vector Machine
WBC: White Blood Cell
WHO: World Health Organization
